# Purpura

**Published:** 1889-10-19

**Authors:** 


### PURPURA.

Dr. J. F. Knott, Diplomate in State Medicine, University of Dublin, publishes a paper on "Heredity in Purpura Hæmorrhagica."† Two cases of fatal epiotaxis are reported occurring in a father and son, accompanied by ecchymotic patches on various parts of the body. The bleeding from the nose resisted everything medical skill could devise, and the coagulative power of the blood was so diminished that it soaked through compressed lint. Dr. Knott observes that the cases present several points of interest with regard to diagnosis, pernicious tendency, and heredity. The latter, as shown by a similar train of symptoms in father and son, involves a complication in the diagnosis, as it might lead some to think of hœmophilia, the hereditary tendency of which is usually much more pronounced. But the absence of any preceding indication of "love of bleeding" decided the diagnosis, which was "morbus maculosis" of Werlhoff, more familiarly known as *purpura hœmorrhagica*. Now, bleeding from the nose often arises from a scorbutic condition of system, and so do ecchymotic patches. Dr. Knott does not say anything on this subject, but it would have been more satisfactory had the author mentioned whether or not there was any scorbutic taint; and as a scorbutic taint may be latent—*i.e.*, not defined by any of the usual signs—it would be desirable to know if the sufferers had been recently exposed to any of the causes of scurvy, especially to a lack of vegetable or fresh meat diet. Again, bleeding from the nose may result from secondary syphilitic nasal affection, which is not mentioned in the report of the cases.

† *Transactions* Royal Academy of Medicine, Ireland, 1889.

